# Isolation and Characterization of New 24 Microsatellite DNA Markers for Golden Cuttlefish (*Sepia esculenta*)

**DOI:** 10.3390/ijms13011154

**Published:** 2012-01-20

**Authors:** Yanjiao Yuan, Shufang Liu, Cuicui Bai, Hongbo Liu, Zhimeng Zhuang

**Affiliations:** 1 Ocean University of Shanghai, Shanghai 201306, China; E-Mail: jiaoyy1987@163.com; 2 Yellow Sea Fisheries Research Institute, Chinese Academy of Fishery Sciences, Qingdao 266071, China; E-Mails: baicuicui77@163.com (C.B.); liuhongbo23@163.com (H.L.); zhuangzm@ysfri.ac.cn (Z.Z.); 3 Ocean University of Dalian, Dalian 116023, China

**Keywords:** golden cuttlefish, *Sepia esculenta*, microsatellite, population structure

## Abstract

Twenty-four microsatellite DNA markers were isolated and characterized for golden cuttlefish (*Sepia esculenta*) from a (GT)_13_—enriched genomic library. Loci were tested in 48 individuals from Jiaozhou bay of China. The numbers of alleles per locus ranged from two to 25 with an average of 10.3. The observed and expected heterozygosities ranged from 0.063 to 0.896 and from 0.137 to 0.953, with averages of 0.519 and 0.633, respectively. Six loci significantly deviated from Hardy-Weinberg equilibrium after Bonferroni’s correction and no significant linkage disequilibrium between loci pairs was detected. These microsatellite markers would be useful for analyzing the population genetic structure to make conservation and management decisions for *S. esculenta*.

## 1. Introduction

Golden cuttlefish (*Sepia esculenta*) is a cephalopod that lives in neritic and abyssal zones, and sometimes in sandy habitats. It is widely distributed, from the island of Honshu in Japan to Vietnam and the Philippines. The golden cuttlefish is an economically important target species in China, South Korea and Japan [1–3]. Unfortunately, since the 1980s, *S. esculenta* resources have been gradually declining possibly due to over-exploitation and ocean environmental change, especially deterioration of the spawning grounds [3]. With respect to the four traditional main spawning grounds of *S. esculenta* along the coast of China, only one remains [4].

Estimating population genetic structure may lead to a better understanding of the effects of over-exploitation and environmental change on *S. esculenta* stocks, and the result would provide valid reference for the management and conservation of fishery resources [5–8]. Among molecular markers, microsatellite markers have been shown to be an extremely valuable tool for the study of population structure because of their variability, abundance, neutrality, co-dominance and unambiguous scoring of alleles [9,10]. Until now, 31 microsatellite markers have been reported for *S. esculenta* [6,8]. However, only 25 of these conformed to the Hardy-Weinberg equilibrium (HWE). Therefore, additional highly informative microsatellite markers are needed for investigating the population genetic structure.

## 2. Results and Discussion

The number of alleles per locus ranged from two to 25, with an average of 10.3. The observed and expected heterozygosities ranged from 0.0625 to 0.8958 and from 0.1366 to 0.9533, with an average of 0.519 and 0.633, respectively (Table 1). Six loci (J5/J8/J17/J61/J63/J74) deviated from HWE in the tested population after Bonferroni’s correction (adjusted *P*-value < 0.002), which might be caused by the heterozygotes deficit. There are three possible explanations for the heterozygotes deficit. Firstly, it might be due to allelic “dropouts”, which are artifacts in the PCR amplification process. Secondly, the deficit might be caused by the limited sample size or size homoplasy. Thirdly, it could result from the presence of null alleles [11,12]. Twenty four microsatellite loci were tested for the presence of null alleles and the results showed that they might be present in seven loci (J5/J8/J10/J17/J60/J63/J74), five of which deviated from HWE. According to Zheng’ report, a significant deviation from HWE was observed in one locus, and null allele existed in the same locus [6]. Wang also reported that, significant occurrences of null alleles were found for four of five loci that deviated from HWE [8]. No significant linkage disequilibrium (LD) was found between all pairs of these 24 loci after Bonferroni’s correction (*P-*value > 0.002).

## 3. Experimental Section

### 3.1. DNA Extraction

Forty-eight individuals of *S. esculenta* were collected from Jiaozhou bay and preserved in alcohol until DNA extraction. DNA was extracted from muscle tissue using the phenol-chloroform procedure [13].

### 3.2. Microsatellite-Enriched Library Construction

Genomic DNA was simultaneously digested with *Mse* I for three hours (New England Biolabs, USA), and the digested DNA (10 μL) was ligated to *Mse* I adaptors (100 pmol) (5′-TACTCAGGAACTCAT-3′/5′-GACGATGAGTCCTGAG-3′). Linker-ligated DNA was amplified in a 25 μL reaction mix using the adapter-specific primer (5′-GATGAGTCCTGAGTAA-3′). Polymerase chain reaction (PCR) conditions were as follows: 20 cycles at 94 °C for 30 s, 53 °C for 1 min, 72 °C for 1 min. The PCR products were purified using DNAmate (TaKaRa, Japan) and hybridized to a biotin labeled (GT_13_ probe. The mixture was denatured at 94 °C for 5 min, then at 53 °C for 15 min. The hybrids were captured with streptavidin-coated magnetic beads (Promega, USA). Unhybridized DNA was washed away, and the remaining DNA was eluted from the magnetic beads and amplified using the adaptor-specific primer and the above PCR program. Following purification, DNA fragments ranging from 500 base pair to 1000 bp were selected by separation on 1.5% agarose gels. The fragments were ligated to pMD18-T vectors (TaKaRa), and transformed into *Escherichia coli* DH5α competent cells to construct an enriched microsatellite library. After amplifying with (GT)_10_ and M13 primers,180 positive clones were obtained. The positive clones were sequenced on an ABI 3730 automated DNA sequencer (Applied Biosystems, USA).

### 3.3. PCR Amplification and Genotyping

Eighty-five pairs of primers were designed using PRIMER PREMIER5 (Premier Biosoft International, USA) and tested for polymorphism with six *S. esculenta*. After preliminary screening, only 24 polymorphic microsatellite loci were tested on a sample of 48 individuals. PCR for all loci was performed separately in a 25 μL reaction volume containing 0.4 μM of each primer, 0.2 mM dNTPs, 2 mM MgCl_2_, 1× PCR buffer, 1 U Taq polymerase (Fermentas, Canada) and 50–100 ng DNA. Amplification was carried out with the following thermal profile: 94 °C for 5 min, followed by 35 cycles of 94 °C for 45 s, optimal annealing temperature (Table 1) for 45 s, and 72 °C for 45 s, and a final extension step at 72 °C for 10 min. PCR products were separated on 6% denaturing polyacrylamide gels and visualized by silver-staining.

### 3.4. Genetic Data Analysis

Allele sizes were estimated according to the pBR322/*Msp* I marker. The variability at each locus was measured in terms of number of alleles, expected heterozygosity and observed heterozygosity, and Hardy-Weinberg equilibrium (HWE) and linkage disequilibrium were tested using GENEPOP 4.0 [14]. Null allele frequencies were calculated using Micro-Checker 2.2.3 [15]. The significant value for all diversity tests of significance was corrected by the sequential Bonferroni’s procedure [16].

## 4. Conclusions

In the present study, we isolated and characterized 24 polymorphic microsatellite loci for *S. esculenta.* These new high variable microsatellite markers will enrich *S. esculenta* microsatellite marker resources and be useful for various population genetic analyses of *S. esculenta*. In our results, there are some highly heterozygous microsatellite loci (*i.e.*, J13/J14/J35/J77), which we will use for designing conservation strategies for the management of *S. esculenta* stocks.

## Figures and Tables

**Table 1:** Characterization of 24 microsatellite loci in 48 *S. esculenta* individuals.

Loci	Genbank accession No.	Repeat motif	Primer sequence(5′-3′)	Size range (bp)	*T*_a_ (°C)	*N*_A_	*H*_O_	*H*_E_	*P*_HW_
J1	JQ317936	(TG)_7_	F: GGTCCAAAGTATGTGAAG R: GTGAAAATGTTGGGTTAT	242–245	55	4	0.2917	0.3535	0.0263
J3	JQ317937	(TG)_9_	F: TCCTCAATCCAAGTCGCTAT R: AACCCAGACACTGATGGTAATC	343–360	55	8	0.3958	0.7627	0.0086
J5	JQ317938	(AC)_11_(AC)_8_(AT)_8_	F: ACGTTTATAAGAGCAACAC R: CAGAATAGATTACCCACAA	217–260	57	18	0.6087	0.9200	0.0000*
J6-1	JQ317939	(TA)_5_	F: GCATCAAAACATAAATAC R: ACTCACTACGAGAAATCA	309–330	55	5	0.4894	0.5468	0.2773
J6-2	JQ317939	(GT)_6_	F: GAATAATTACTCAGAGGCAC R: TCTATTTCCATTTTCGTG	320–350	57	4	0.5455	0.5541	0.0474
J8	JQ317940	(CA)_27_	F: ATTTCAGTTATGGTCCTTG R: ATTCTGTAGCCATCAAGC	300–380	57	13	0.7083	0.8789	0.0006*
J10	JQ317941	(AC)_6_..(AC)_23_(ATC)_5_	F: CAGCCTCACTACGAAGAA R: RGATACGCAACCGAGACAC	300–330	45	5	0.3404	0.5269	0.0161
J12	JQ317942	(TG)_9_	F: CGTTTGCGTAGGATGTCA R: GTCGGTCTTGGTACTTTCAC	250–260	55	4	0.6250	0.6656	0.1807
J13	JQ317943	(TC)_9_(TC)_6_(CT)_7_(GTAT)_7_(TATC)_7_	F: TAAGTTTCGTAGGGTATCAC R: ATGTATTTCGGCTTTGGA	290–380	55	17	0.8085	0.8943	0.0152
J14	JQ317944	(CA)_12_	F: CAAGCAGGATTCAAGTTC R: TTTATCATCATTCCCAGG	250–290	55	21	0.8958	0.9349	0.0913
J17	JQ317945	(TG)_25_	F: ATTGGAAATCGGTGAGCT R: GATGGGAGTTGGGAAATG	217–260	55	20	0.7234	0.8950	0.0000*
J19	JQ317946	(TG)_16_	F: ACTAGCTTAGCTGGAGACG R: GAAATTGGCTTGGTGATT	217–250	50	9	0.5227	0.6084	0.0265
J25	JQ317947	(AC)_9_	F: CACATGGCCTAAAGATTG R: AAGGGTGGAGAATAGTTTG	195–205	55	6	0.6458	0.6401	0.6535
J35	JQ317948	(AC)_21_	F: GAGAAGCGACAAGCAATA R: GACTGTAACCTGGAAGCA	220–270	57	19	0.8511	0.9272	0.0026
J48	JQ317949	(GT)_6_	F: GCAAACCAATAGGTCATC R: TACTTGTACCGAAAAGCA	270–290	57	5	0.4894	0.6436	0.0137
J50	JQ317950	(TG)_6_..(GA)_5_	F: TGTTCCGTGTCGCCTTTG R: TGGGTCGTTGGACAACCTG	217–230	55	4	0.2558	0.3425	0.0063
J58	JQ317951	(GT)_10_	F: AGACCCAGTAGTAAGCAAA R: TCCACTAACTTGGAGCAT	264	55	3	0.5106	0.6111	0.0110
J60	JQ317952	(TG)_5_	F: AATTTCGATCATCTTCCAT R: CATTCCAATAGACATTTTGTA	190	55	2	0.0625	0.1366	0.0116
J61	JQ317953	(AG)_27_	F: CACAATGTATACTACGCCTCT R: ATGCTTACCTCTTTATCCG	240–300	55	20	0.8750	0.9419	0.0000*
J63	JQ317954	(AC)_5_(AC)_13_	F: GAAAACGATACAAGGAGT R: GTGCAAGAAACAAAGACA	260–309	55	13	0.6042	0.8936	0.0000*
J74	JQ317955	(AC)_26_	F: GTTGGGAAATGCAAAGTC R: CAAGTTACAGCGGAGAAA	242–309	52	25	0.8085	0.9533	0.0000*
J77	JQ317956	(CT)_5_(CA)_18_	F: TTCCTCACAAACTATTCT R: TGATTTCTCCATCTGTTA	310–400	55	10	0.6809	0.8055	0.1457
J78	JQ317957	(AC)_9_	F: TGTGAACCCGAAACGAAC R: ATGGCAAGGAGAATGTGG	310–360	50	5	0.4894	0.7376	0.0024
J83	JQ317958	(TG)_7_	F: TAAGCAAGACCAGAGTAGCC R: GTAAATTGTTGTGCAAATCC	250–280	50	7	0.7391	0.8321	0.1032

*T*_a_, annealing temperature (°C); *N*_A_, number of alleles; *H*_O_, observed heterozygosity; *H*_E_, expected heterozygosity;

* indicated deviation from Hardy-Weinberg equilibrium (*P* < 0.05) after Bonferroni’s correction; *P*_HW_, Hardy-Weinberg probability test.
